# Trial and error: evaluating and refining a community model of HIV testing in Australia

**DOI:** 10.1186/s12913-017-2635-z

**Published:** 2017-10-10

**Authors:** Kathleen E. Ryan, Alisa Pedrana, David Leitinger, Anna L. Wilkinson, Peter Locke, Margaret E. Hellard, Mark Stoové

**Affiliations:** 1 Centre for Population Health, Burnet Institute, Commercial Road, Melbourne, VIC 3004 Australia; 2 School of public health and preventive medicine, Monash University, Commercial Road, Melbourne, VIC 3004 Australia; 3 Department of Nutrition, T.H. Chan School of Public Health, Harvard University, Huntington Avenue, Cambridge, MA 02115 USA; 4 PRONTO!, Victorian AIDS Council, Rose St, Fitzroy, VIC 3065 Australia; 5 Infectious Disease Department, Alfred Hospital, Commercial Road, Melbourne, VIC 3004 Australia

**Keywords:** Community service, HIV testing, Acceptability, Barriers, Msm, Australia, Service refinement

## Abstract

**Background:**

The 2012 regulatory approval of HIV rapid point of care (RPOC) tests in Australia and a national strategic focus on HIV testing provided a catalyst for implementation of non-clinical HIV testing service models. PRONTO! opened in 2013 as a two-year trial delivering peer-led community-based HIV RPOC tests targeting gay, bisexual and other men who have sex with men (GBM), with the aim of increasing HIV testing frequency. Initial data suggested this aim was not achieved and, as part of a broader service evaluation, we sought to explore client acceptability and barriers to testing at PRONTO! to refine the service model.

**Methods:**

We present descriptive and thematic analyses of data from two in-depth evaluation surveys and four focus groups with PRONTO! clients focused on service acceptability, client testing history, intentions to test and barriers to testing for HIV and other sexually transmitted infections (STIs).

**Results:**

The three novel aspects of the PRONTO! model, testing environment, rapid-testing, peer-staff, were reported to be highly acceptable among survey and focus group participants. Focus group discussions revealed that the PRONTO! model reduced anxiety associated with HIV testing and created a comfortable environment conducive to discussing sexual risk and health. However, an absence of STI testing at PRONTO!, driven by restrictions on medical subsidies for STI testing and limited funds available at the service level created a barrier to HIV testing. An overwhelming majority of PRONTO! clients reported usually testing for STIs alongside HIV and most reported plans to seek STI testing after testing for HIV at PRONTO!. When deciding where, when and what to test for, clients reported balancing convenience and relative risk and consequences for each infection as guiding their decision-making.

**Conclusions:**

A community-based and peer-led HIV testing model reduced previously reported barriers to HIV testing, while introducing new barriers. The absence of STI testing at PRONTO! and the need to access multiple services for comprehensive sexual health screening, created a significant service engagement barrier for some clients. Understanding client motivations to access testing and ensuring novel service models meet client needs is crucial for developing acceptable sexual health services for high-risk populations.

## Background

In 2012 the Australian Therapeutic Goods Administration (TGA) approved the first HIV rapid point-of-care (RPOC) test device for use in Australia [1]. Alongside a renewed strategic emphasis on HIV testing [2], this approval provided a catalyst for change in HIV testing services locally, including testing in non-clinical settings and by people trained in use of the device. Gay, bisexual and other men who have sex with men (GBM) are the key HIV priority population in Australia, and based on previous research [3, 4] it was anticipated that peer-led RPOC testing undertaken in a community-based service would reduce barriers and encourage men to test more frequently.

HIV testing is an integral component of HIV prevention strategies globally. While HIV testing provides an opportunity for risk reduction counselling and prevention education, the strategic prevention emphasis on testing lies with its place as the essential first step in the treatment cascade [2]. Timely HIV diagnosis, a result of frequent testing, facilitates early access to HIV treatment and viral suppression, offering significant benefit both to an individual [5] and the community through the prevention of onward transmission [6]. HIV testing is emphasised in Australia’s National HIV Strategy [2] and in global HIV prevention advocacy [7]. Modelling suggests that HIV transmission is disproportionately driven by undiagnosed HIV [8–10]. Australian modelling has suggested that increased testing frequency to quarterly testing is likely to have the greatest (albeit modest) impact on reducing transmission [11].

Local and international HIV testing guidelines are risk-based [12–14]; in Australia all sexually active GBM are recommended to test for HIV and other sexually transmissible infections (STIs) annually, with testing up to four times per year recommended for men classified as ‘high-risk’ [14]. Clinic-level assessments of HIV testing patterns suggest sub-optimal HIV testing rates among the majority of Australian GBM [15, 16]. The majority of HIV testing in Australia occurs in primary care clinical settings and, prior to the approval of RPOC tests, GBM reported that more convenient forms of HIV testing, including rapid testing, community testing and home/self-testing, would increase their testing frequency [3, 11, 17, 18].

Although models of RPOC testing may reduce known barriers to testing, rigidity within the Australian universal healthcare system, Medicare, limit the expansion of testing for HIV and STIs through non-conventional service delivery models. Specifically, the majority of laboratory-based HIV and other STI tests are rebated through Medicare, however application for listing of a HIV RPOC test on the Medicare Benefits Schedule was unsuccessful, and thus no rebate is provided for HIV RPOC tests [19]. Secondly, whilst RPOC test device registration in Australia permits their use by trained peer staff outside traditional clinical settings [1], only registered medical professionals (and not trained peers) can request Medicare rebated pathology [20]. This regulatory environment limits the range of tests available in a peer delivered HIV RPOC testing service, specifically those related to confirmatory HIV serological tests and tests for other STIs.

In the context of restricted Federal funding and increased prevention efforts, a number of Australian jurisdictions provided funding for HIV RPOC testing services for GBM [21–23]. RPOC testing is currently offered within clinical services (as user-pays models), and in stand-alone fixed site and outreach/pop-up community services funded by jurisdictional governments, with services delivered ranging from comprehensive primary healthcare to stand-alone HIV RPOC tests. Internationally, it is well established that RPOC testing services are highly acceptable [24], but little Australian evidence of acceptability exists. Moreover, few studies have examined the impact of RPOC testing services on HIV and STI test frequency, and whether these services continue to present barriers to testing [24, 25].

As part of an evaluation of PRONTO!, a community-based and peer-led RPOC HIV testing service for GBM, we previously reported that during the first 18 months of operations the proportion of GBM returning for a HIV test within 6 months was no better than that observed at clinical services in Melbourne [26]. Here we use mixed-methods evaluation data to explore the acceptability of this service model and examine any continued barriers to HIV testing.

## Method

### The service

PRONTO! operations have been described in detail elsewhere [26]. Briefly, PRONTO! began operations on 15 August 2013 in an inner-city location in Melbourne under management of the Victorian AIDS Council and funding from the Victorian Department of Health and Human Services to provide free HIV RPOC tests to Victorian GBM. Novel aspects of PRONTO! (in the context of the Australian HIV testing models) include operating outside normal business hours, testing delivered by peer staff, a shop-front community-based setting and the use of a RPOC test. Funding barriers precluded the integration of STI testing, with symptomatic clients referred to clinical services.

During a 30-min appointment peer staff deliver pre- and post-test counselling and perform a HIV RPOC test using the Trinity Biotech Unigold HIV 1/2 test device as per manufacturer’s instructions [27]. During the 10 min test incubation clients remain in the room with the peer test facilitator, during which time clients are routinely engaged in a sexual health discussion. The RPOC test result is then delivered in the same consultation. Follow up is based on RPOC test result, including confirmatory testing for all reactive results.

### The evaluation

A mixed-methods evaluation was employed to assess the trial aims; to reduce barriers to HIV testing, increase HIV testing frequency, and increase access to sexual health services among GBM. The evaluation included integration of PRONTO! as a participating site in an existing sentinel surveillance system of high HIV caseload clinics. Additionally, in-depth evaluation surveys, and client focus group discussions (FGD) were conducted periodically and key informant interviews were conducted at the mid trial point. PRONTO! managers and staff were consulted during the development of all data collection tools and evaluation reports were distributed to the program managers and funders annually [28, 29]. To explore model acceptability and factors contributing to decisions to test for HIV and STIs we present data from sentinel surveillance, two evaluation surveys and four FGDs.

### Sentinel surveillance and evaluation surveys

#### Recruitment and data collection

Sentinel surveillance data collection has been described elsewhere [26]. Briefly, at each appointment clients complete a brief survey containing demographics, testing history and sexual risk characteristics. At the first appointment, clients are assigned a unique numerical identifier which facilitates the linking of survey and test data at each appointment and over time.

For PRONTO! evaluation surveys, a consecutive sampling method was employed. All clients testing between 12 November 2013 and 31 January 2014 (survey one) and between 15 May 2014 and 05 October 2014 (survey two) were invited to complete surveys by peer staff. No financial reimbursement was provided to clients completing survey one and clients completing survey two were compensated $20 for their time. The surveys contained sections on demographics, HIV and STI testing history, barriers and motivators to testing, acceptability of the PRONTO! model, sexual risk, and marketing and communications.

#### Data analysis

All clients who self-identified as male, reported any male-to-male sex and/or self-identified as gay or bisexual, and were aged 18 years and over were included in the analysis.

To assess the representativeness of the evaluation survey samples, selected items (age, country of birth, region of residence and sexual risk classification (high/not high risk based on national risk based testing guidelines, [14]) asked on both sentinel surveillance and evaluation surveys were compared using test of proportions. We describe the acceptability of the service model (accessibility, reception and physical environment, rapid testing process, and peer-led model), and compare these outcomes over time (surveys one and two) using a two sample z-test.

To explore HIV and STI testing behaviours and intentions we describe HIV and STI testing history (if ever tested for HIV, time since last HIV test, location of last HIV test, usually test for HIV and STIs together) and intentions to test (plans to seek STI testing following most recent test at PRONTO! and likeliness to return to PRONTO! for testing). Additional variables added to survey two were included in the descriptive analyses to explore preference for peer staff, barriers to HIV and STI testing and recent testing history (if tested for HIV or STI elsewhere since testing at PRONTO!, reason for test, and location of test).

Quantitative data were analysed using *Stata Statistical Software: Release 13* (StataCorp, College Station, TX: StataCorp LP) and the cut-off for statistical significance was *p* < .05 for all analyses.

### Focus group discussions

#### Recruitment and data collection

All evaluation survey participants were invited to provide contact details for participation in FGDs. All clients who provided contact details (survey one: *n* = 47, survey two: *n* = 189) were invited by email to participate in a FGD. Two FGDs were held at the Burnet Institute following each survey round (four in total) lasting approximately 90 min each. FGD themes included motivators and barriers to testing, acceptability of the service, structural, social and community benefits of the service, and service marketing; in this paper we include FGD data related to service acceptability and factors associated with decisions to test for HIV and STIs. FGDs were audio recorded and transcribed verbatim. Pseudonyms were assigned to all FGD participants.

#### Data analysis

Following the second round of FGDs, all focus group data were pooled and thematically analysed by KR. FGD analysis was performed using NVivo 11.

### Ethics and consent

The Alfred Hospital Ethics approved the evaluation of the RPOC testing service (HREC 297/13) and use of the test device in a clinical trial (261/13). Inclusion of the service as a site in the sentinel surveillance system was approved by the Victorian Department of Health Ethics Committee (52/05).

For clients completing the behavioural sentinel surveillance survey, consent is implied with completion of the survey, as previously detailed elsewhere [30]. For clients completing the evaluation surveys and participating in any FGDs, all participants completed a participant’s informed consent form prior to participation.

## Results

### Evaluation surveys

Over the 24-month trial period, 1947 GBM attended PRONTO! and received 2998 RPOC HIV tests. During evaluation survey one and two recruitment periods, 229 and 737 clients, respectively, tested for HIV at PRONTO!, met the eligibility criteria and were invited to participate in surveys. Of these, 118 (52% response rate) and 298 (40% response rate) GBM completed survey one and two, respectively. There were no significant differences in the characteristics of clients attending PRONTO! during the trial period (as determined from sentinel surveillance surveys) and clients completing the evaluation surveys (Table 1).

**Table 1:** Characteristics of PRONTO! GBM clients completing sentinel surveillance, evaluation survey one, and survey two

	Sentinel surveillance^a^	Survey one		Survey two	
	n (%)	n (%)	*p*-value^b^	n (%)	*p*-value^c^
	1947 (100)	118 (100)		298 (100)	
Age					
18–29	935 (48.0)	55 (46.6)	0.75	133 (44.6)	0.37
30–39	613 (30.5)	39 (33.1)	0.75	94 (31.5)	0.86
40+	399 (20.5)	24 (20.3)	0.98	71 (23.8)	0.53
Median(IQR)	30.45 (26–38)	30 (26–36)		31 (26–39)	
Country of birth					
Australia	1094 (58.1)	79 (68.1)	0.10	192 (66.0)	0.06
Other	789 (41.9)	37 (31.9)	0.25	99 (34.0)	0.16
Residence^d^					
Metro Melbourne	823 (93.7)	110 (93.2)	0.22	288 (96.6)	0.56
Regional Victoria	30 (3.4)	2 (1.7)	0.86	6 (2.0)	0.80
Other/missing	25 (2.9)	6 (5.1)		4 (1.3)	
High Risk^e^					
No	619 (31.8)	31 (26.3)	0.50	108 (36.2)	0.40
Yes	1328 (68.2)	87 (73.7)	0.26	190 (63.8)	0.26

^a^Recorded at clients first test at PRONTO!, missing excluded

^b^Two sample z-test, samples include sentinel surveillance and survey one

^c^Two sample z-test, samples include sentinel surveillance and survey two

^d^Not asked in sentinel surveillance in first year of operations

^e^Any condomless anal sex and/or more than 10 anal sex partners in 6 months

#### Service acceptability

Clients completing survey one reported high acceptability across almost all aspects of the model, with an overwhelming majority of clients reporting that they were likely to return to PRONTO!. Acceptability of most aspects of the model remained high in survey two. While there were significant reductions in the proportion of the samples reporting they ‘preferred rapid testing to conventional HIV testing’, or ‘found the conversation [with peer test facilitators] useful in regards to my sexual health’ from survey one to two, these aspects of the model remained highly acceptable among survey two participants (>80% agreement with statements). A significant decline was observed in preferences for testing with a peer compared to sexual health doctor or nurse; peers were preferred by approximately three quarters of respondents in survey one, decreasing to under two thirds in survey two (Table 2).

**Table 2:** Acceptability of novel aspects of the PRONTO! model among PRONTO! GBM clients completing evaluation survey one and survey two

	Survey one n (%)^a^ 118 (100.0)	Survey two n (%)^a^ 298 (100.0)	*P*-value^b^
The opening hours are convenient for me			
Any agree	111 (94.1)	264 (88.6)	
Not agree	7 (5.9)	34 (11.4)	0.09
The location of PRONTO! is convenient for me			
Any agree	94 (79.7)	227 (76.4)	
Not agree	24 (20.3)	70 (23.6)	0.48
I found it easy to get an appointment at PRONTO!			
Any agree	117 (99.2)	287 (96.6)	
Not agree	1 (0.9)	10 (3.4)	0.15
I was comfortable waiting in the consultation room with the test facilitator			
Any agree	111 (94.1)	291 (97.7)	
Not agree	7 (5.9)	7 (2.3)	0.07
Overall, I prefer rapid testing to conventional HIV testing			
Any agree	116 (98.3)	261 (87.6)	
Not agree	2 (1.7)	37 (12.4)	0.001
I found our conversation useful in regards to my sexual health			
Any agree	109 (92.4)	250 (83.9)	
Not agree	9 (7.6)	48 (16.1)	0.02
They managed the whole experience professionally			
Any agree	118 (100.0)	293 (98.3)	
Not agree	0 (0.0)	5 (1.7)	0.16
Overall, I prefer testing with a peer test facilitator rather than a sexual health doctor or nurse			
Any agree	92 (78.0)	179 (60.3)	
Not agree	26 (22.0)	119 (35.4)	0.001
I am likely to return to PRONTO! for HIV testing			
Any agree	113 (95.8)	279 (93.9)	
Not agree	5 (4.2)	18 (6.1)	0.46
I am likely to test for HIV more frequently now that PRONTO! is open			
Any agree	101 (85.6)	254 (85.2)	
Any disagree	17 (14.4)	44 (14.8)	0.9

Any agree – agree, strongly agree; not agree – neither agree nor disagree, disagree, strongly disagree

^a^Missing excluded

^b^two sample z-test

To explore factors contributing to a lower reported preference for peers relative to other acceptability indicators, survey two respondents could respond in free text to the question, ‘why you did or did not prefer testing with a peer compared to sexual health doctor or nurse’. Almost one third (*n* = 88) of respondents provided comment. Among those reporting no preference for peer (*n* = 119), 35 provided a free text response to describe their preference for peer or medical staff. Two reasons were provided by respondents for preferring a doctor or nurse; an increased comfort in receiving a positive result from a doctor or nurse and concern with confidentiality in a peer delivered service. However, comments most commonly (*n* = 16) highlighted the acceptability of HIV testing delivered by either peer staff or doctors nurses, with respondents preferring a service that was “professional and confidential” with preference depending on “the person, not their job title”.

#### HIV and STI testing within the PRONTO! Model

Most PRONTO! evaluation survey participants were already engaged in routine HIV testing in clinical settings prior to attending PRONTO!. Approximately two thirds of those with a HIV testing history reported frequent (previous test within 6 months) HIV testing and around half reported usually testing at a sexual health centre, with smaller proportions testing at high HIV caseload general practitioner (GP) clinics. Almost all of these participants reported usually testing for STIs at the same time as HIV. After testing at PRONTO!, three quarters of survey one respondents reported they ‘planned to seek STI testing following their most recent test at PRONTO!. This proportion declined to approximately half at survey two. There was no significant difference in the age, country of birth or high risk classification among those who did and did not plan to seek STI testing (data not shown). The most common location for planned STI testing was a sexual health centre or high HIV caseload clinic, consistent with participants’ usual HIV testing routine (Table 3).

**Table 3:** HIV and STI test history of PRONTO! GBM clients completing evaluation survey one and survey two

	Survey one n (%)^f^ 118 (100)	Survey two n (%)^f^ 298 (100)	*p*-value^g^
Ever tested for HIV			
No	15 (13.3)	48 (16.2)	
Yes	98 (86.7)	248 (83.3)	0.50
Typical HIV testing frequency^a^			
Every 6 months or less	66 (67.4)	147 (59.5)	0.28
Every 12 months	25 (25.5)	52 (21.1)	0.66
Every 24 months or more	7 (7.1)	48 (19.4)	0.43
Usual testing service^a^			
Sexual health centre	47 (48.0)	93 (37.8)	0.28
High HIV caseload GP clinic	19 (19.4)	57 (23.2)	0.75
Another GP	20 (20.4)	56 (22.8)	0.84
Other	8 (8.2)	18 (7.3)	0.75
No usual service	4 (4.1)	22 (8.9)	0.98
Usually test for HIV and STIs at the same time^a^			
No	4 (4.4)	25 (10.1)	0.11
Yes	87 (95.6)	222 (89.9)	0.72
Do you plan to seek STI testing following your most recent test at PRONTO!			
No	28 (24.8)	140 (47.8)	0.03
Yes	85 (75.2)	153 (52.2)	< 0.01
Where do you plan to have this test^b^			
Sexual health centre	50 (58.1)	61 (40.4)	0.06
High HIV caseload clinic	15 (17.4)	50 (33.1)	0.24
Another GP	14 (16.3)	32 (21.2)	0.70
Other	7 (8.1)	8 (5.3)	0.82
When do you plan to attend for this STI test^b^			
In the next month	47 (54.7)	68 (45.0)	0.31
In the next 3 months	28 (32.6)	58 (38.4)	0.60
In the next 3-12 months	10 (11.6)	22 (14.6)	0.83
In 12 months or more	1 (1.2)	3 (2.0)	–
Since your first test at PRONTO! have you tested for HIV at a different clinic^c^			
No		215 (74.4)	
Yes		74 (25.6)	
Where did you have this test ^c d^			
Sexual health centre		23 (31.1)	
High HIV caseload clinic		26 (35.1)	
Another GP		17 (23.0)	
Other		8 (10.8)	
Please indicate why ^c d e^			
I wanted to test for all STIs, including HIV		52 (65.8)	
I had a doctor’s appointment for other reasons and I asked for or was offered an HIV test at the same time		12 (16.0)	
I prefer to include conventional HIV tests as part of my testing routine		9 (12.0)	
I received a reminder to test from my usual service		4 (5.3)	
I had already booked an appointment for sexual health testing		3 (4.0)	
I prefer testing at my usual HIV testing service		1 (1.3)	
Since your first test at PRONTO!, have you tested for STIs other than HIV at a different clinic^c^			
No		208 (70.5)	
Yes		87 (29.5)	

^a^Of those who have previously tested for HIV

^b^Of those who plan to seek STI testing

^c^Survey two only

^d^Of those who reported testing elsewhere since their first test at PRONTO! (*n* = 74)

^e^Multiple selections allowed

^f^Missing excluded.

^g^-Two sample z-test.

Additional questions were added to survey two to explore clients decision to test for HIV &/or STIs at other services since first testing at PRONTO!. One quarter of the survey two respondents reported testing for HIV elsewhere since first testing at PRONTO!, two thirds of whom reported that they did so to access comprehensive STI testing. Almost one third of survey two respondents reported testing for STIs at another clinic since first testing at PRONTO! (Table 3).

### Focus group discussion

Twenty six men participated in one of four FGDs. The age range across the four FGDs was 20-46 years and most had tested for HIV before attending PRONTO! for the first time.

Focus group discussions covered a range of evaluation themes, however we have restricted analysis to acceptability of the PRONTO! model and factors that contributed to a clients’ decision to test for HIV &/or STIs.

#### Testing environment

The physical space at PRONTO! was described as “contemporary without being too medical” and this was reported as improving clients’ test experience. Participants referred specifically to certain physical and sensory elements of the PRONTO! reception space, such as the couch, low reception desk, large window and the music playing as an “inviting” environment compared to previous experiences at clinical services.

*“I expected it to be like a doctors clinic, you walk in and get take a ticket, you’re told to sit on an uncomfortable chair, you sit there and wait for your name to be called out whereas like the guy was having a chat, gave me a clipboard, take a seat on the couch, help yourself to a drink, like, this is pretty cool” Paul, 28 years*

#### Rapid testing process

Many participants discussed the rapid testing process and immediate results delivery as an improvement to the client experience. Participants spoke about their anxiety around testing for HIV which was somewhat allayed by the service at PRONTO!, in particular by the reduced time to receiving their HIV test result.

*“I’m generally not ever that worried about it (HIV), as I said it’s only when I’ve just got the test done that you start… the mind starts ticking, and the rapid test reduces that to 10 minutes and it means that I’m quite happy to go far more rather than if I had to wait a week every time” Evan, 25 years*

While some participants reported having concerns about rapid test device accuracy and performance compared to conventional testing prior to attending PRONTO!, all agreed that their confidence in the technology improved following their experience at the service. Nevertheless, some participants reported that they would maintain occasional testing at clinical services because of the higher sensitivity of laboratory testing and peace of mind in accessing what they felt was more reliable laboratory-based HIV testing.

*“I guess I was reassured of, you know, I got to ask lots of questions about it and I felt reassured that it was just as effective as the standard test”* Gabe, 25 years

#### Peer staff model

The benefits of the peer model largely centred around a feeling that clients could have meaningful conversation on sexual health and broader issues pertinent to GBM, rather than be “lectured” on sexual risk. The 10 minute device incubation offered structured time and the opportunity for discussion between client and peer staff, which was discussed by most participants as a useful time to ask questions they would normally not ask a doctor or nurse. Participants reported this opportunity was generally not available at clinical services due to time and staffing constraints. Many participants commented that testing with a peer and the increased opportunity for dialogue was something that they did not expect when first attending PRONTO!, while a few preferred to wait outside the room, most appreciated this aspect of the PRONTO! model.

*“the fact that you’ve got that wait time where you’re having a conversation with them waiting for the test results… you certainly don’t do that with a GP, a GPs mostly just sitting at their computer typing … but there was greater structured time for an actual conversation” Lawrence, 44 years*

The value ascribed to the peer discussion was enhanced by the relationship that clients built with the peer staff. Participants described the improved test experience with a more relatable person conducting the test compared to participants’ previous experience with doctors and/or sexual health nurses. Some men discussed feeling at ease knowing that it was a peer testing them, and felt that peer testers broke down barriers that existed in standard clinical dialogue, enabling them to be more open in discussion about their sexual health.

*“(the peer environment) lightens the mood, that’s for sure… that brings down barriers to discuss things that maybe bothering you or at the back of your mind” Vinay, 36 years*

A number of participants weren’t aware that PRONTO! was a peer-led service when they first attended, and concerns were raised by some participants about confidentiality and potentially being tested by someone they knew. These concerns seemed to be allayed by the level of professionalism displayed by peer test facilitators. The professional conduct of the peer staff, balanced with the general relaxed style of the service, was important to participants feeling confident in the RPOC test result.

*“I didn’t feel at all uncomfortable with them not being professionals and they did explain that they have received the training to do this test” Evan, 25 years*

#### Convenience of the PRONTO! Model

During FGDs, participants discussed factors that influenced their motivation and decision to test for both HIV and STIs, including convenience, and their perceived relative risk and seriousness of certain infections. During these discussions, many participants reflected on the importance of convenient models of testing offered by PRONTO!. Men described the model as conducive to fitting HIV testing into their life. The ability to book an appointment after business hours with minimal planning and to receiving their test result without a return visit was highlighted.

*“It made it less of a chore, like I never thought getting a test could be that easy, I just made an appointment half an hour before I was meeting my friends to go get burgers at Fitzroy, and I got it done and walked down Smith St and met them and had dinner” Ben, 20 years*

#### Testing for HIV contrasted with testing for other STIs

When discussing how well the services met the health needs of men, the lack of STI testing offered at PRONTO! was highlighted as a major barrier. In this context men highlighted the need to attend other services for comprehensive STI testing as a major inconvenience reducing the likelihood they would return to PRONTO!, regardless of perceiving the service model as otherwise highly acceptable service.

*“Just because I don’t want to go to two places, I would probably just go to my one place and get everything done in one sitting” Hugo, 21 years*

Participants differed in their perceptions of their need to access STI testing in addition to HIV testing; these perceptions appeared to influence their decision to return to PRONTO! or test for HIV and STIs elsewhere. Some participants discussed their perceived need for HIV and STI testing in the context of a hierarchy of consequence, with a HIV diagnosis having the most severe consequence and conferring the greatest need for testing. Participants making testing decisions on this basis would delay asymptomatic STI testing at other clinical services in preference for more convenient HIV testing at PRONTO!.

*“HIV’s the big concern, but anything else there’s either symptoms or… depending on what’s going on, if you think you’ve had any exposure to anything, so that (no STI testing) wouldn’t affect my using the service” Ulric, 26 years*

Other participants discussed their decision to test for HIV and STIs in the context of a hierarchy of perceived risk of infection. These participants generally spoke about the higher likelihood of acquiring an STI other than HIV resulting in a preference for comprehensive HIV and STI screening.

*“I probably won’t use PRONTO!, for me, I’ll just go back to the (sexual health clinic) ‘cause, you know, HIV is just one test… it’s not something that bugs me, it’s the others that bug me more” Vinay, 36 years*

## Discussion

The key features of the PRONTO! service model, peer delivered RPOC testing in a non-clinical setting, were highly acceptable to clients, however the absence of STI testing in the model provided a major disincentive for clients to return to PRONTO! for HIV testing. Depending on client preferences and sexual health priorities, the inability to offer STI testing alongside convenient RPOC HIV testing has significant implications for HIV and STI prevention and care. Those electing to return and use PRONTO! as their routine access point to HIV testing will ultimately miss altogether or delay asymptomatic STI testing. Those reporting not returning because they are accessing STI testing elsewhere miss out on the otherwise acceptable and convenient aspects of the model and may not test for HIV any more frequently than they did before. In either case, primary aims of the service model are not achieved. Consistent with evaluations of community-based and/or RPOC testing services internationally [31], we found an overwhelmingly high level of acceptability across many defining aspects of the PRONTO! model. The high acceptability observed in this study is consistent with other Australian studies reporting high acceptability of RPOC testing in clinical settings [22, 32]. However, relative to PRONTO! testing environment and the provision of rapid testing, the peer staffing model was the least acceptable aspect of the three key domains. While we observed modest declines in acceptability ratings across some service domains between surveys one and two, reported preference for peer testers compared to a sexual health doctor or nurse declined more notably from 78% to 60%. We have previously reported that survey one respondents who did not prefer peers were more likely to report having a majority of gay friends, testing at PRONTO! for reasons other than it being a gay friendly service and reporting not learning anything new about HIV during their appointment [29]. As many of the benefits of the peer model reported in the FGDs centred around increased comfort with staff and opportunity for discussion and education, the peer model may offer fewer benefits for men who are gay community attached and well informed about sexual health. The high acceptability of specific components of the PRONTO! peer model (e.g., comfort with peer, professionalism of peers) is juxtaposed with a lower preference for peer testers compared to doctors or nurses. This may simply indicate that peer involvement is acceptable to clients but not a primary contributing factor for why some men to choose to test at or return to PRONTO!. Alternately, the findings presented in this paper suggest the benefits of a peer-led model may depreciate with multiple attendances at PRONTO! as clients’ perceived need for peer-led education and counselling diminishes with multiple exposures.

While the PRONTO! service model was acceptable to an overwhelming majority of clients, the lack of STI testing offered at PRONTO! meant that this model may have inadvertently introduced new barriers to testing that may to disrupt clients’ routine HIV/STI testing patterns and negatively impact their HIV and STI testing frequency. Prior to testing at PRONTO! an overwhelming majority (91%) of survey respondents reported usually accessing comprehensive STI testing alongside HIV testing, however, accessing comprehensive testing since attending PRONTO! was less commonly reported and diminished over time; only 50% of survey two respondents reported they planned to seek STI testing following their most recent HIV test at PRONTO!. This suggests that despite peer staff encouraging clients to access comprehensive testing elsewhere this was not a priority for a substantial proportion of clients. It was clear from focus group discussions that clients’ decision on where, when and what to test for were driven by multi-faceted individual hierarchies that included convenience, self-perceived risk of acquisition, and the consequence of specific infections. The influence of these factors on clients’ decision to test for HIV/STIs may contribute to delaying or not returning for HIV testing at PRONTO! or delaying accessing STI testing at a clinical service. In either case, by reducing men’s opportunity to access comprehensive HIV/STI testing at one service, the PRONTO! testing model appeared to work against the stated aims of the service to increase HIV testing frequency and increase access to sexual health services. This is of particular concern when considered in the context of a marked increase in syphilis and *Neisseria gonorrhoea* notifications among GBM locally over the same period [33]. A service that contributes, albeit unintentionally, to a reduced frequency of STI testing and misses opportunities for opportunistic screening is concerning. In the context of restrictive funding and regulatory barriers that maintain a central role for clinicians in HIV/STI testing, many benefits of patient-centred community-based models of health care may be undone.

The issues raised in this paper relate to broader regulatory barriers to the implementation of adapted and tailored models of care. For example, the lack of regulatory approval in Australia for point-of-care STI tests means that convenient and acceptable testing models for STIs are not readily delivered to high-risk populations in regional and remote Australia, such as those recently trialled in Northern Australia. The further development and funding for non-conventional models of care including point-of care tests, and hybrid community-based and clinical models is needed to offer clients highly acceptable and convenient models of comprehensive HIV and STI testing.

In response to the evaluation findings presented here, the Victorian AIDS Council (service managers) funded and implemented a *Chlamydia trachomatis* and *Neisseria gonorrhoea* testing pilot to explore the acceptability and feasibility of integrated STI testing at PRONTO!. Three-quarters of clients attending for HIV tests during the trial period opted to also receive STI testing and a high proportion of clients tested positive for STIs (19% tested positive for at least one STI). However, delays in result delivery, inability to prescribe treatment and absence of sustainable funding (partly driven but restrictions in public subsidies for clinician oversight) suggested that incorporating STI testing into the existing PRONTO! model was not feasible [34]. PRONTO! management have since amended the service model, including engaging general practitioners to work onsite, primarily in a trans and gender diverse clinic and HIV pre-exposure prophylaxis clinic, offering support to STI testing and treatment among PRONTO! clients. In this way, PRONTO! management has developed a model that maintains both the community-based features and the convenience of RPOC testing at PRONTO!, while enabling a sustainable funding structure for comprehensive STI testing. Preliminary analysis of repeat testing at PRONTO! following the introduction of STI testing shows a significant increase in 6 month repeat testing [35] and additional research into the impact of this change is ongoing.

There are a number of limitations in this study. Recruitment of participants to the evaluation surveys and the FGDs may be biased. To assess selection bias we compared demographics and sexual risk reported in each evaluation survey with that reported in PRONTO! behavioural sentinel surveillance and found no significant differences between the samples. Recruitment into the FGDs may have been biased as these participants went through two levels of self-selection. In addition, survey responses and FGDs may be influenced by social desirability bias. Previous work has shown a discrepancy between high self-reported frequent testing and lower objective testing data from clinics [16]; as such, self-reported data on typical HIV and STI testing frequencies may have been biased towards more frequent testing.

## Conclusions

While free RPOC HIV testing in a community-based, peer-led service was highly acceptable to clients, this did not translate to increased HIV testing frequency during the trial period [26]. Evaluation data presented here suggests that the siloed HIV testing model employed at PRONTO! during the trial period was likely to have contributed to a lack of impact on HIV test frequency. Understanding the motivations and circumstances for why and when men seek HIV and STI testing and ensuring service models are implemented with these considerations in mind, is important to address the continued suboptimal test frequency among high risk GBM.

## Abbreviations

FGD: Focus group discussion
GBM: Gay, bisexual and other men who have sex with men
RPOC: Rapid point-of-care
STI: Sexually transmissible infection
TGA: Therapeutic Goods Administration
